# ﻿The psyllid genus *Ciriacremum* (Hemiptera, Psyllidae) in Brazil

**DOI:** 10.3897/zookeys.1258.167703

**Published:** 2025-11-03

**Authors:** Daniel Burckhardt, Dalva Luiz de Queiroz

**Affiliations:** 1 Naturhistorisches Museum, Augustinergasse 2, 4001 Basel, Switzerland Naturhistorisches Museum Basel Switzerland; 2 Embrapa Florestas, Estrada da Ribeira, Km 111, CP. 319, CEP 83411-000, Colombo, PR, Brazil Embrapa Florestas Colombo Brazil

**Keywords:** Ciriacreminae, Fabaceae, jumping plant lice, Neotropics, Psylloidea, Sternorrhyncha, taxonomy

## Abstract

*Ciriacremum* is a genus of Ciriacreminae (Hemiptera, Sternorrhyncha, Psylloidea, Psyllidae) comprising 26 validly described species in the Afrotropical realm and only one in the Neotropics. Here we add four new species from Brazil, which are named and diagnosed, including illustrations and an identification key for adults: *Ciriacremum lanceolatum***sp. nov.** (from Mato Grosso), *C. maederae***sp. nov.** (from Amazonas), *C. pollicigerum***sp. nov.** (from Amazonas) and *C. roraima***sp. nov.** (from Roraima). Two other species are reported from Brazil (Amazonas and Pará) but not formally named due to insufficient material. Apart from *C. setosum* (from Nicaragua) and the Brazilian species treated in this paper, there are at least four additional undescribed species from the Neotropics (Colombia, Guyana, Panama, Suriname, and Venezuela), which are briefly discussed. Little is known about the immatures and host plants of the Neotropical species. The only confirmed host is *Hymenaea* sp. (Fabaceae) of *C. maederae***sp. nov.** Adults were collected also on other fabaceous genera that are possible hosts. In the Neotropics, *Ciriacremum* is restricted to the tropics between Nicaragua in the north and Brazil (Mato Grosso) in the south.

## ﻿Introduction

Psyllids or jumping plant lice (Hemiptera, Sternorrhyncha, Psylloidea), which feed exclusively on plant sap, are generally very host-specific insects. While in the northern hemisphere many genera occur in the Nearctic and Palaearctic realms (e.g. *Aphalara* Foerster, *Bactericera* Puton, *Cacopsylla* Ossiannilsson, *Craspedolepta* Enderlein, *Livia* Latreille, *Psylla* Geoffroy, *Spanioneura* Foerster, and *Trioza* Foerster) and several genera have a pantropical distribution (e.g. *Calophya* Löw, *Colophorina* Capener, *Diclidophlebia* Crawford, *Euryconus* Aulmann, *Melanastera* Serbina, Malenovsky, Queiroz & Burckhardt, and *Pseudophacopteron* Enderlein) (Hodkinson 1980, 1989; Ouvrard 2020; Burckhardt et al. 2024), there is only one genus that is restricted to the Afrotropical and Neotropical realms: *Ciriacremum* Enderlein (Hodkinson 1989).

The Afrotropical species of *Ciriacremum* were revised by Hollis (1976), who recognised 23 species. Aléné et al. (2007) added 13 species from Gabon. Six species described from Cameroon are not valid, as no holotypes were selected (Yana et al. 2021). Only one described species, *Ciriacremum setosum* Crawford from Nicaragua, is known from the Neotropics (Crawford 1914). Hodkinson and White (1981) reported *C. setosum* from Guyana based on material deposited in the Natural History Museum, London (NHMUK). Unidentified *Ciriacremum* species were also reported from Panama (Brown and Hodkinson 1988), Venezuela (Aléné et al. 2007), Brazil (Burckhardt and Queiroz 2012), and Colombia (Rendón-Mera et al. 2017). A single specimen from Brazil is also photographed in iNaturalist (https://www.inaturalist.org/observations/221943814).

**Figure 1.**
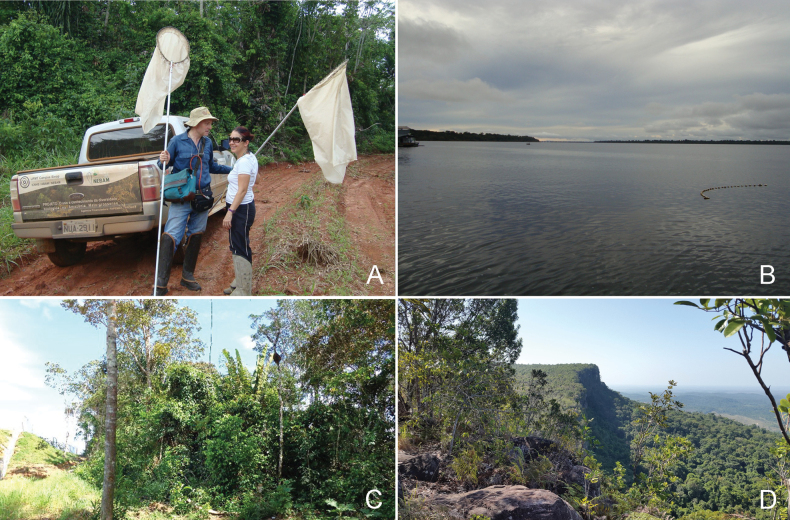
Habitats of *Ciriacremum* spp. in Brazil. A. Amazonian forest in MT with the two authors while collecting psyllids; B. Parque Nacional de Anavilhanas, AM with view on the wide expanse of the Rio Negro; C. detail of forest in AM; D. Serra do Tepequém, RR.

Fieldwork carried out over the last 15 years in various Brazilian states (Fig. 1) with the aim of improving knowledge of the diversity and biology of psyllids has revealed four *Ciriacremum* species, all of which are unnamed and whose taxonomy is the subject of the present paper.

## ﻿Materials and methods

The Brazilian material was collected by D. Burckhardt, D.L. de Queiroz, and collaborators in, or is reported from, the following states: Amazonas (**AM**), Mato Grosso (**MT**), Pará (**PA**), Roraima (**RR**). The material examined or cited is deposited in the following institutions: Muséum d’histoire naturelle, Geneva (**MHNG**), Switzerland; Museum für Naturkunde der Humboldt Universität Berlin (**MNHU**), Germany; Naturhistorisches Museum, Basel (**NHMB**), Switzerland; The Natural History Museum (**NHMUK**), London, U.K.; Coleção Entomológica Padre Jesus Santiago Moure, Centro Politécnico, Universidade Federal do Paraná (**UFPR**), Curitiba, PR, Brazil. The plant vouchers were identified by M.L. Brotto and J.T.W. Motta (Museu Botânico Municipal, Curitiba, PR). They are deposited at the NHMB.

The morphological terminology follows Bastin et al. (2023), and that the forewing venation and the male terminalia accords with Hollis (1976). The plant names follow Plants of the World (POWO 2025).

## ﻿Taxonomy

### ﻿ Ciriacremum

Taxon classificationAnimaliaHemipteraPsyllidae

Enderlein, 1910

C5D1D89F-13DB-5BEC-95AE-BC6E66D9F47A

Ciriacremum Enderlein, 1910: 139. Type species: Ciriacremum filiverpatum Enderlein, by original designation.
Ceriacremum ; Crawford (1914): 63; incorrect subsequent spelling.
Bunoparia Enderlein, 1926: 397. Type species: Ciriacremum capillicorne Enderlein, by original designation. Synonymised by Hollis (1976): 24.

#### Description.

A detailed description of the genus is provided by Hollis (1976). Based on the male terminalia Hollis (1976) defined two species groups in the Afrotropical region, but leaving several species ungrouped. He did not discuss the Neotropical *Ciriacremum setosum*. The Neotropical species can be diagnosed as follows.

##### ﻿The *Ciriacremum setosum* Crawford species group

**Figure 2.**
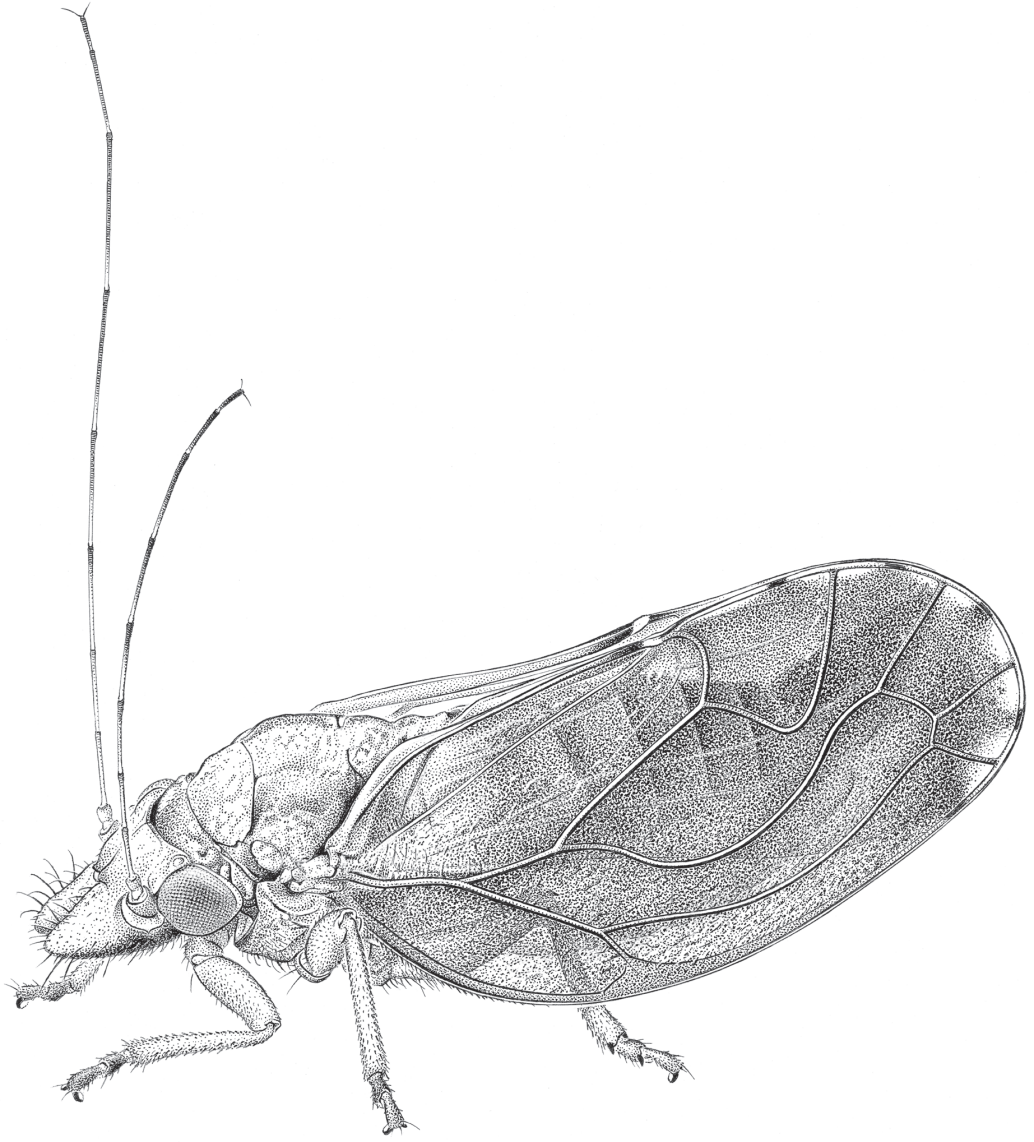
Habitus in lateral view of *Ciriacremum maederae* sp. nov. (drawing A. Coray).

**Figure 3.**
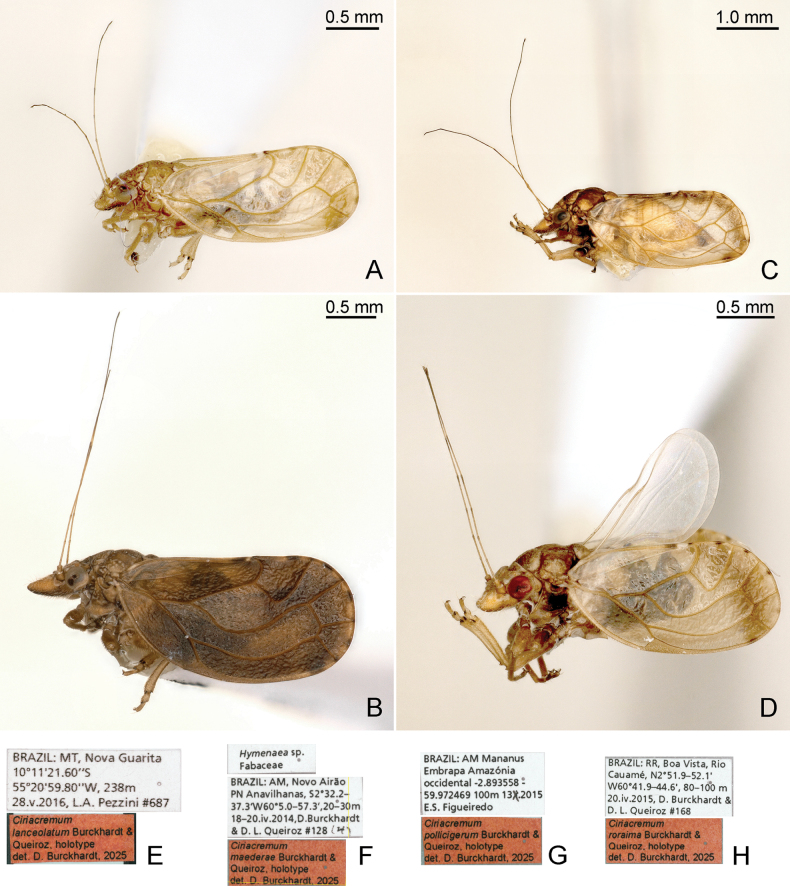
Habitus in lateral view (A–D) and holotype labels (E–H) of *Ciriacremum* spp. A, E. *Ciriacremum lanceolatum* sp. nov.; B, F. *Ciriacremum maederae* sp. nov.; C, G. *Ciriacremum pollicigerum* sp. nov.; D, H. *Ciriacremum roraima* sp. nov. Scale bars: 0.5 mm (A, B, D); 1.0 mm (C).

**Figure 4.**
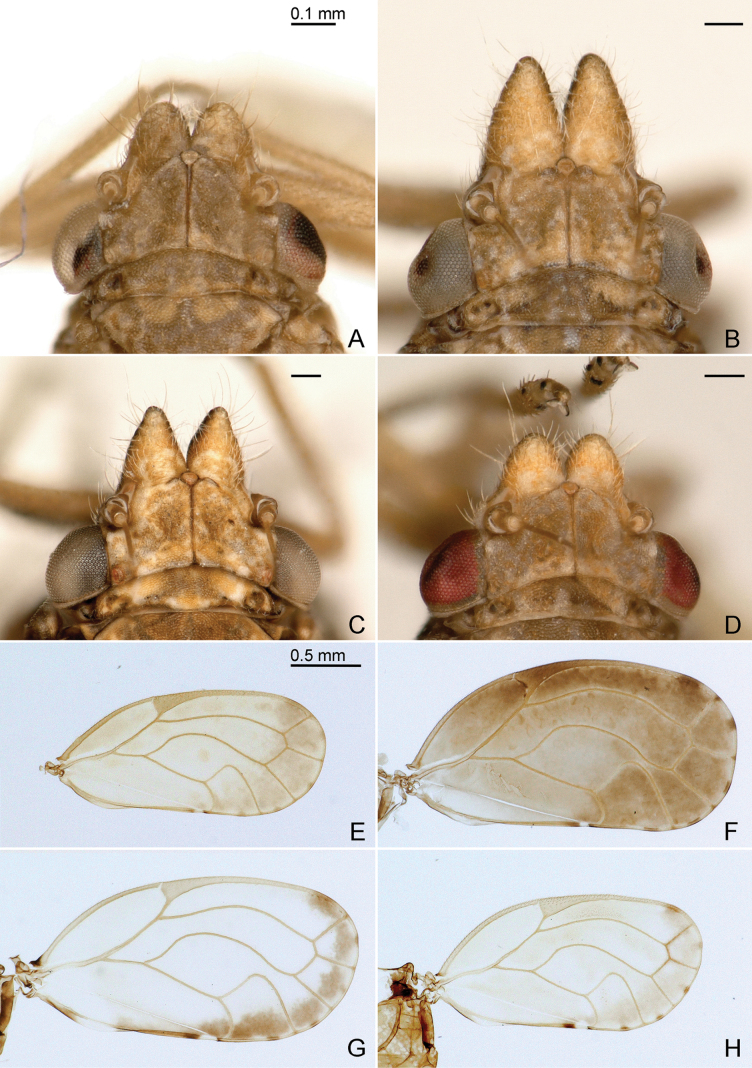
Head in dorsal view (A–D) and forewing (E–H) of *Ciriacremum* spp. A, E. *Ciriacremum lanceolatum* sp. nov.; B, F. *Ciriacremum maederae* sp. nov.; C, G. *Ciriacremum pollicigerum* sp. nov.; D, H. *Ciriacremum roraima* sp. nov. Scale bars: 0.1 mm (A–D); 0.5 mm (E–H).

**Figure 5.**
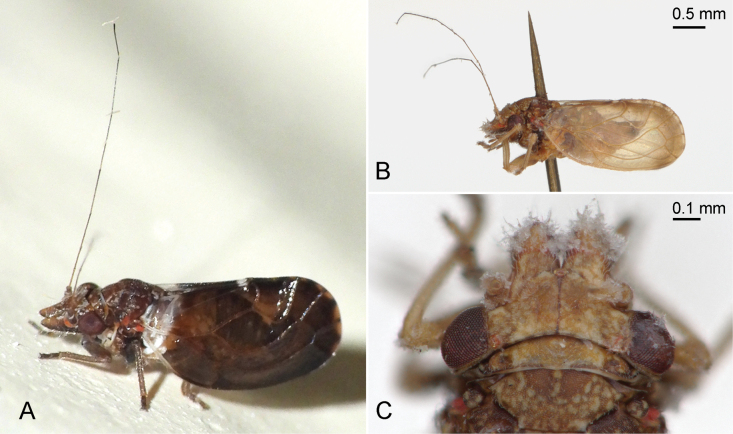
Habitus in lateral view (A, B) and head in dorsal view (C) of *Ciriacremum* spp. A. *Ciriacremum* sp. A, Pará (photo S. Dantas; iNaturalist https://www.inaturalist.org/observations/221943814); B, C.*Ciriacremum* sp. B, Amazonas (photos L.Š. Serbina). Scale bars: 0.5 mm (B); 0.1 mm (C).

**Figure 6.**
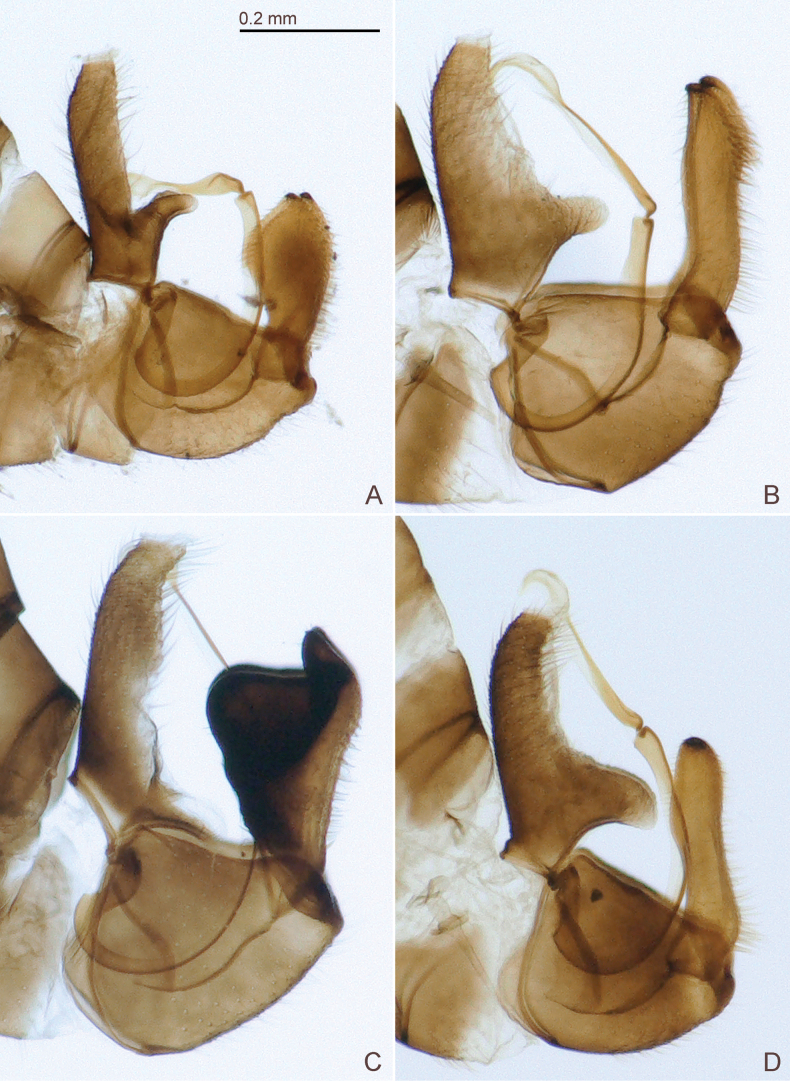
Male terminalia of *Ciriacremum* spp., in lateral view. A. *Ciriacremum lanceolatum* sp. nov.; B. *Ciriacremum maederae* sp. nov.; C.*Ciriacremum pollicigerum* sp. nov.; D. *Ciriacremum roraima* sp. nov. Scale bar: 0.2 mm.

**Figure 7.**
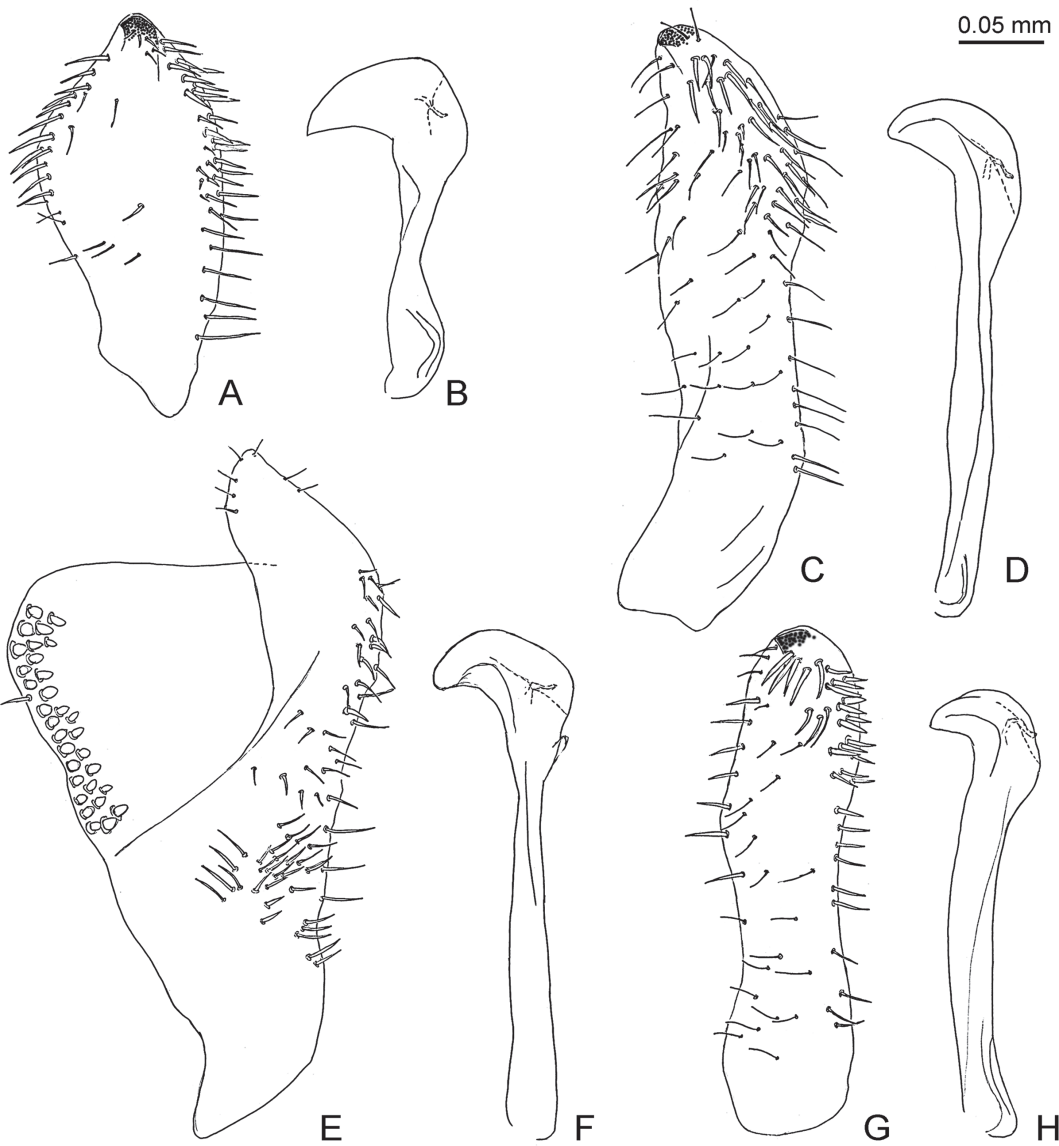
Inner face of paramere, in lateral view (A, C, E, G), and distal segment of aedeagus, in lateral view (B, D, F, H) of *Ciriacremum* spp. A, B. *Ciriacremum lanceolatum* sp. nov.; C, D. *Ciriacremum maederae* sp. nov.; E, F. *Ciriacremum pollicigerum* sp. nov.; G, H. *Ciriacremum roraima* sp. nov. Scale bar: 0.05 mm.

**Figure 8.**
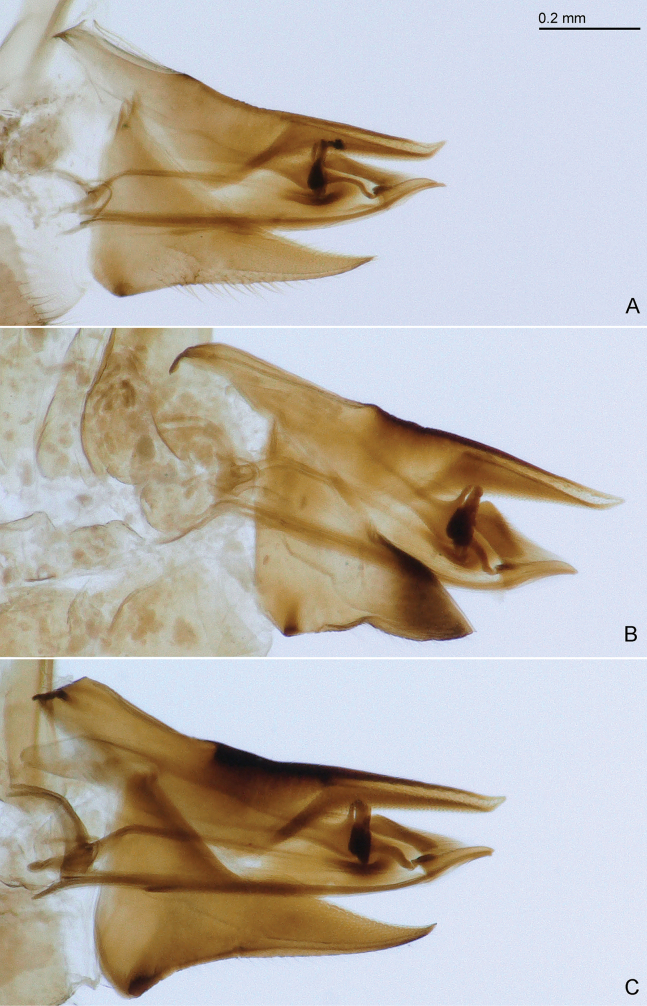
Female terminalia of *Ciriacremum* spp. A. *Ciriacremum maederae* sp. nov.; B. *Ciriacremum pollicigerum* sp. nov.; C.*Ciriacremum roraima* sp. nov. Scale bar: 0.2 mm.

**Diagnosis. Adult** (Figs 2, 3A–D, 5A, B). Head, in lateral view, weakly inclined (ca 30°) from longitudinal body axis (Figs 2, 3A–D, 5A, B); in dorsal view, wider than pronotum, about as wide as mesoscutum (Figs 4A–D, 5C). Vertex subtrapezoidal, relatively flat including the area along coronal suture; anteorbital tubercle small, flattened; genal processes conical, 0.7–1.1 times as long as vertex along coronal suture, pointed or blunt apically; compound eyes moderately large, hemispherical, slightly recessive; preocular sclerite absent; in lateral view, distance between hind margin of occiput and forewing base about the same as or less than diameter of eye. Rostrum short, 0.2–0.4 times as long as head width. Antenna 2.4–3.5 times as long as head width; segments VII or VIII longer than III. Propleurites narrow; episternum and epimeron subequal. Parapteron subglobular, much larger than tegula. Metapostnotum with moderately prominent median, longitudinal ridge. Metatibia with prominent genual spine and with 1+3+1 apical spurs. Forewing (Fig. 4E–H) ellipsoid to subrhomboid, 2.4–3.0 times as long as head width, 1.9–2.1 times as long as wide; pterostigma short, pedunculate, r-m cross vein long; surface spinules, densely spaced, covering membrane up to the veins; radular spinules present in the middle along the margins of following cells: m_1_, m_2_ and cu_1_. Hindwing with distinctly grouped costal setae. Male proctiger (Fig. 6) with posterior lobes; subgenital plate subglobular without hypovalves (Fig. 6); distal segment of aedeagus (Fig. 7B, D, F, H) long, tubular with hook-shaped apical dilatation. Female terminalia (Fig. 8) long, cuneate; proctiger 1.0–1.2 times as long as head width, with a group of long lateral setae in basal third, a submedian longitudinal row of long setae on either side and densely spaced peg setae in apical two-thirds; circumanal ring oval, consisting of two unequal rows of pores; dorsal valvula cuneate, long and narrow, weakly curved, ventral valvula weakly curved.

**Fifth instar immature.** Unknown.

**Affinities.** The species of the *Ciriacremum setosum* group differ from those of the Afrotropical realm by the presence of densely arranged surface spinules of the forewings, which cover the membrane up to the veins (exceptions *C. julbernadoides* Hollis and *C. orientale* Hollis). Most African species possess hypovalves at the rear end of the male subgenital plate (absent in *C. capense* Enderlein, *C. capilicorne* Enderlein, *C. filiverpatum* Enderlein, and *C. gabonense* Aléné, Hoess & Burckhardt) and lack a posterior lobe on the male proctiger (exception *C. capense*), which is always present in the New World species. In many African species the genal processes are short; in the Neotropical species they are always longer than 0.5 times the vertex length along the coronal suture. Adults of the Brazilian species can be identified with the following key.

### ﻿Key to the Brazilian *Ciriacremum* species

**Table:** 

1	Genal processes longer than 0.8 times vertex along coronal suture, pointed or subacute apically (Figs 4B, C, 5C). AM, PA	**2**
–	Genal processes shorter than 0.8 times vertex along coronal suture, relatively blunt apically (Fig. 4A, D). MT, RR	**5**
2	Forewing dark (Figs 2, 3B, 5A). AM, PA	**3**
–	Forewing light with distinct or indistinct dark brown apical band (Figs 3C, 4G, 5B). AM	**4**
3	Forewing dark also at base (Fig. 3B). Terminalia as in Figs 6B, 7C, D, 8A. AM	***Ciriacremum maederae* sp. nov.**
–	Forewing with white transverse band at base (Fig. 5A). PA	***Ciriacremum* sp. A**
4	Forewing with well-defined, dark, transverse apical band (Figs 3C, 4G). Terminalia as in Figs 6C, 7E, F, 8B	***Ciriacremum pollicigerum* sp. nov.**
–	Forewing with ill-defined, dark, transverse apical band (Fig. 5B)	***Ciriacremum* sp. B**
5	Antenna long, 2.9 times head width. Forewing with R_1_ short, about one-third as long as maximum width of pterostigma (Fig. 4E). Paramere lanceolate, shorter than male proctiger (Figs 6A, 7A); distal segment of aedeagus with large apical dilatation (Fig. 7B). Female unknown. MT	***Ciriacremum lanceolatum* sp. nov.**
–	Antenna short, 2.4 times head width. Forewing with R_1_ long, about as long as maximum width of pterostigma (Fig. 4H). Paramere lamellar, longer than male proctiger (Figs 6D, 7G); distal segment of aedeagus with small apical dilatation (Fig. 7H). Female terminalia as in Fig. 8C. RR	***Ciriacremum roraima* sp. nov.**

### ﻿ Ciriacremum lanceolatum

Taxon classificationAnimaliaHemipteraPsyllidae

Burckhardt & Queiroz sp. nov.

C9432F78-3E3D-5AF3-8E90-38E6A8C18821

https://zoobank.org/8C54F8D2-321A-40DB-9245-F78BD777885B

Figs 3A, 4A, E, 6A, 7A, B

#### Type locality.

Brazil, Mato Grosso state, Nova Guarita municipality, forest at the Rio Peixoto de Azevedo River 15 km NE of Nova Guarita, 10.1893°S, 55.3499°W.

#### Type material.

***Holotype*.** Brazil • ♂; MT, Nova Guarita; 10.1893°S, 55.3499°W; 240 m a.s.l.; 28 May 2016; L.A. Pezzini leg.; sweeping; #687; UFPR, slide-mounted (Fig. 3E).

#### Description.

**Adult male** (Fig. 3A). Colour. Overall colour brown. Vertex greyish brown; genal processes slightly lighter; antennae yellowish, segments III–VIII with dark apices, segments IX and X dark brown. Mesoscutum with four longitudinal, narrow brown stripes. Legs yellowish, metafemur dark brown. Forewing transparent with light-brown band apically, radular areas brown; veins yellowish. Abdominal tergites yellow, sternites and male proctiger almost black; male subgenital plate and parameres light brown.

***Structure*.** Genal processes 0.6 times as long as coronal suture, blunt apically (Fig. 4A). Antenna 2.9 times as long as head width, relative length of flagellar segments from base to apex as 1.0: 0.8: 1.2: 1.4: 1.7: 1.6: 0.8: 0.4. Metatibia 0.7 times as long as head width. Forewing (Figs 3A, 4E) 2.8 times as long as head width, 2.1 times as long as wide; evenly rounded apically. Terminalia as in Figs 6A, 7A, B. Proctiger 0.4 times as long as head width, slender in apical two-thirds, posterior lobe curved, pointing caudad. Paramere shorter than proctiger; in lateral view, lanceolate; subacute apically; inner face with a row of bristles along fore margin and a band of bristles along hind margin; with small sclerotised hook apically. Distal segment of aedeagus much shorter than proctiger; with large apical dilatation.

***Measurements*** (1 ♂, in mm). Head width 0.72; antenna length 2.10; forewing length 2.00; male proctiger length 0.32; paramere length 0.28; length of distal portion of aedeagus 0.20.

**Adult female** and **fifth instar immature** unknown.

#### Etymology.

From the Latin adjective *lanceolatus*, “lanceolate”, referring to the shape of the paramere in lateral view.

#### Distribution.

Brazil (MT).

#### Host plant.

Unknown.

#### Affinities.

A member of the *Ciriacremum setosum* group, *C. lanceolatum* sp. nov. shares the short, apically blunt genal processes with *C. roraima* sp. nov., from which it differs as indicated in the key. The antennae are longer, the vein R_1_ of the forewing is shorter, the paramere is lanceolate rather than lamellar and the apical dilatation of the aedeagus is larger. From *C. setosum* it differs in the slightly smaller body dimensions (head width 0.72 mm versus 0.91 mm in *C. setosum*, forewing length 2.00 mm versus 2.10 mm), the shorter and apically blunt genal processes (about as long as coronal suture and apically subacute in *C. setosum*); the lanceolate paramere (with large anterior lobe in *C. setosum*).

### ﻿ Ciriacremum maederae

Taxon classificationAnimaliaHemipteraPsyllidae

Burckhardt & Queiroz sp. nov.

18960B51-1DD8-5C0E-8121-1B7E8349037A

https://zoobank.org/D7B3C250-82C7-4903-8ADC-6B66690B437D

Figs 2, 3B, 4B, F, 6B, 7C, D, 8A

#### Type locality.

Brazil, Amazonas state, Novo Airão municipality, Anavilhanas National Park, 2.5366°S/2.6217°S, 60.8327°W/60.9559°W.

#### Type material.

***Holotype*.** Brazil • ♂; AM, Novo Airão, Parque Nacional de Anavilhanas; 2.5366°S/2.6217°S, 60.8327°W/60.9559°W; 20–30 m a.s.l.; 18–20 Apr. 2014; D. Burckhardt & D.L. Queiroz leg.; *Hymenaea* sp., Amazonas inundation forest; #128(4); UFPR, dry (Fig. 3F).

***Paratypes*.** Brazil • 6 ♂, 11 ♀; same data as holotype; NHMB (NMB-PSYLL0003486, NMB-PSYLL0005730, NMB-PSYLL0009747 to NMB-PSYLL0009752), UFPR, dry, slide-mounted and in 70% ethanol.

#### Other material examined.

(not included in type series) Brazil • 187 immatures of younger instars; same data as holotype; NHMB (NMB-PSYLL0009753), in 70% ethanol.

#### Description.

**Adult** (Figs 2, 3B). Colour. Overall colour dark brown. Head and thorax yellow dorsally; head and genal processes dark brown ventrally; antenna yellow; segments V–VII dark brown apically; segments VIII–X dark brown. Pronotum dark in the middle; mesopraescutum with antero-median brown patch; mesoscutum with two narrow and two broad, longitudinal, dark-brown bands. Pro- and mesofemora brown. Forewing brown, with dark-brown band along anterior margin and brown band along apical margin; in the middle of wing slightly lighter; in the middle of cells along apical margin with brown spots.

***Structure*.** Genal processes 1.0–1.1 times as long as coronal suture, pointed or subacute apically (Fig. 4B). Antenna 3.3–3.5 times as long as head width, relative length of flagellar segments from base to apex as 1.0: 1.2: 1.6: 1.7: 2.2: 2.3: 1.2: 0.4. Metatibia 0.7–0.8 times as long as head width. Forewing (Fig. 4F) 2.7–3.0 times as long as head width, 1.9–2.0 times as long as wide; slightly truncate apically. Terminalia as in Figs 6B, 7C, D, 8A. Male proctiger 0.4–0.5 times as long as head width, slightly conical in apical two-thirds, posterior lobe straight, pointing caudad. Paramere as long as or longer than proctiger; in lateral view, lamellar, slightly expanded along posterior margin in apical third; subacute apically; inner face with long setae in apical two-thirds and a group of long bristles along hind margin in apical third; with small sclerotised hook apically. Distal segment of aedeagus as long as or slightly shorter than proctiger; with moderately large, weakly curved apical dilatation. Female proctiger 1.0–1.1 times as long as head width, apex obliquely truncate, slightly upturned; circumanal ring 0.2–0.3 times as long as proctiger. Subgenital plate 0.6–0.7 times as long as proctiger, bearing dense bristles in apical half.

***Measurements*** (2 ♂, 2 ♀, in mm). Head width 0.76–0.84; antenna length 2.60–2.96; forewing length 2.06–2.44; male proctiger length 0.32–0.38; paramere length 0.36–0.38; length of distal portion of aedeagus 0.32; female proctiger length 0.84–0.86.

**Fifth instar immature** unknown.

#### Etymology.

Noun in the genitive case; dedicated to our dear friend Dr h. c. Felicitas Maeder in recognition of her many years of work as administrator of Pro Entomologia, the foundation that partly financed our collections and studies on Brazilian Psylloidea.

#### Host plant.

*Hymenaea* sp. (Fabaceae).

#### Distribution.

Brazil (AM).

#### Affinities.

A member of the *Ciriacremum setosum* group, *C. maederae* sp. nov. shares the long, apically pointed or subacute genal processes with *C. pollicigerum* sp. nov., *C.* sp. A, and *C.* sp. B, from which it differs as indicated in the key. It differs from *C. pollicigerum* and sp. B in having a mostly dark-brown forewing (versus with a transverse marginal band), and from sp. A in lacking a white forewing base. *Ciriacremum maederae* sp. nov. shares the long, apically pointed genal processes with *C. setosum* from which it differs in the dark-brown forewing (versus bearing a dark marginal band), the lamellar paramere (versus paramere with large anterior lobe) and the relatively longer female subgenital plate (about two-thirds as long as proctiger versus about half as long).

### ﻿ Ciriacremum pollicigerum

Taxon classificationAnimaliaHemipteraPsyllidae

Burckhardt & Queiroz sp. nov.

82848582-8FBD-50A0-A3D6-9C29673B5567

https://zoobank.org/FF2647C9-D1DC-46E6-83AD-DD1110F41924

Figs 3C, 4C, G, 6C, 7E, F, 8B

#### Type locality.

Brazil, Amazonas state, Manaus municipality, Embrapa Amazônia Ocidental research centre, 2.8994°S, 59.9745°W.

#### Type material.

***Holotype*.** Brazil • ♂; AM, Manaus, Embrapa Amazônia Ocidental; 2.8994°S, 59.9745°W; 90–100 m a.s.l.; 13 Oct. 2015; E.S. Figueiredo leg.; cultivated plants; UFPR, dry mounted (Fig. 3G).

***Paratypes*.** Brazil • 1 ♂; same data as holotype; 13 Mar. 2014; C. Krug & E. Soares leg.; *Tachigali paniculata*, cultivated plants; NHMB (NMB-PSYLL0009758), slide-mounted • 1 ♀; same data as holotype; 24 Mar. 2014; E. Soares leg.; cultivated plants; NHMB (NMB-PSYLL0009759); slide-mounted • 1 ♂; same data as holotype; 24 Mar. 2014; E.S. Figueiredo leg.; *Hymenaea courbaril*, #CPAA (01), cultivated plants; NHMB (NMB-PSYLL0009760); dry mounted • 3 ♂ 5 ♀; same data as holotype; NHMB (NMB-PSYLL0005248, NMB-PSYLL0009761), UFPR, dry mounted and in 70% ethanol.

#### Description.

**Adult** (Fig. 3C). Colour. General body colour brown. Vertex ochreous with small yellow dots; head ventrally dark brown. Antennal segments I and II brown, segments IV–VIII with dark apices, segments IX and X dark brown. Pronotum brown with a submedian yellow patch on either side. Mesopraescutum ochreous with small yellow dots; mesoscutum brown with median longitudinal yellow band and small yellow dots, some arranged in four longitudinal rows. Mesepimeron brown. Foreleg and mesofemur brown. Forewing mostly transparent with yellow veins; vein A dark brown; with dark-brown, well-contrasted band along apical margin; margin at radular areas brown. Younger specimens rather yellow with more extended light colour.

***Structure*.** Genal processes 0.9 times as long as coronal suture, subacute apically (Fig. 4C). Antenna 3.2–3.4 times as long as head width, relative length of flagellar segments from base to apex as 1.0: 1.1: 1.3: 1.7: 2.0: 2.1: 1.0: 0.3. Metatibia 0.6–0.7 times as long as head width. Forewing (Fig. 4G) 2.5–2.7 times as long as head width, 2.0–2.1 times as long as wide; broadly rounded apically. Terminalia as in Figs 6C, 7E, F, 8B. Male proctiger 0.4 times as long as head width, mostly tubular, posterior lobe at base, straight, pointing caudad, weakly sclerotised. Paramere longer than proctiger; in lateral view, inversely triangular, with a posterior thumb-like process; inner face with several rows of peg setae along fore margin in the middle, and moderately long setae in posterior half. Distal segment of aedeagus shorter than proctiger; moderately large, with strongly curved apical dilatation. Female proctiger 1.0 times as long as head width, apex subacute; circumanal ring 0.3 times as long as proctiger. Subgenital plate 0.4 times as long as proctiger, bearing moderately dense setae in apical half.

***Measurements*** (1 ♂, 1 ♀, in mm). Head width 0.96–1.02; antenna length 3.28–3.30; forewing length 2.40–2.80; male proctiger length 0.38; paramere length 0.42; length of distal portion of aedeagus 0.30; female proctiger length 1.00.

**Fifth instar immature** unknown.

#### Etymology.

From the Latin noun *pollux*, “thumb”, and the Latin suffix -*ger*, “bearing”, referring to the thumb-like process on the paramere in lateral view.

#### Host plant.

Unknown. Adults were collected on *Hymenaea courbaril* and *Sclerobium paniculatum* (Fabaceae) which are possible hosts.

#### Distribution.

Brazil (AM).

#### Affinities.

A member of the *Ciriacremum setosum* group, *C. pollicigerum* sp. nov. shares the long, apically pointed or subacute genal processes with *C. maederae* sp. nov., *C.* sp. A, and *C.* sp. B, from which it differs as indicated in the key. It differs from *C. maederae* and *C.* sp. A in having a transverse marginal band on the forewing (versus mostly dark brown or black), and from *C.* sp. B in having a well-defined marginal band on the forewing rather than an indistinctly delimited one. *Ciriacremum pollicigerum* sp. nov. resembles *C. setosum* in the long, apically pointed genal processes, the apically banded forewing, the anteriorly lobed paramere, and the short female subgenital plate (<0.5 times proctiger length). It differs from the latter species in the slightly larger body dimensions (head width 0.96–1.02 mm versus 0.91 mm, forewing length 2.40–2.80 mm versus 2.1 mm) and details of the paramere (anterior lobe broadly rounded anteriorly with 2–3 rows of peg setae along anterior margin on inner face which is glabrous otherwise versus anterior lobe narrowly rounded anteriorly with bristles densely covering entire inner face).

### ﻿ Ciriacremum roraima

Taxon classificationAnimaliaHemipteraPsyllidae

Burckhardt & Queiroz sp. nov.

E7870F3A-B46A-5FB7-BA29-F004CB9F3E90

https://zoobank.org/470D434A-9C85-4A97-B9C8-A3B88AF46691

Figs 3D, 4D, H, 6D, 7G, H, 8C

#### Type locality.

Brazil, Roraima state, Boa Vista municipality, Rio Cauamé, 2.8650°N/2.8683°N, 60.6983°W/60.7433°W.

#### Type material.

***Holotype*.** Brazil • ♂; RR, Boa Vista, Rio Cauamé; 2.8650°N/2.8683°N, 60.6983°W/60.7433°W; 80 m a.s.l.; 20 Apr. 2015; D. Burckhardt & D.L. Queiroz leg.; degraded riverine forest, #168(-); UFPR, dry mounted (Fig. 3H).

***Paratypes*.** Brazil • 1 ♀; same data as holotype; NHMB (NMB-PSYLL0009755); slide-mounted • 1 ♂; RR; Boa Vista; Embrapa campus; 2.7550°N, 60.7300°W; 80 m a.s.l.; 1–2 Apr. 2015; D. Burckhardt & D.L. Queiroz leg.; *Deguelia densiflora*, #151(3), various experimental plantations, secondary scrub; NHMB (NMB-PSYLL0009756), dry mounted • 1 ♂; RR, Boa Vista, 42 km E Boa Vista, Campo Esperimental Água Boa; 2.6717°N, 60.8400°W; 80 m a.s.l.; 2 Apr. 2015; D. Burckhardt & D.L. Queiroz leg.; degraded Cerrado vegetation, #154(-); NHMB (NMB-PSYLL0009757), in 70% ethanol • 1 ♂; RR, Boa Vista, Praia do Polar; 2.8667°N, 60.6483°W; 80 m a.s.l.; 20 Apr. 2015; D. Burckhardt & D.L. Queiroz leg.; degraded riverine forest, #167(-); NHMB (NMB-PSYLL0009754); slide-mounted.

#### Description.

**Adult** (Fig. 3D). Colour. Overall colour dark brown. Head greyish brown; genal processes yellow dorsally and ventrally; antennae yellowish, segments I and II brown, segments III–VIII with dark apices, segments IX and X dark brown. Thorax light brown with light dots dorsally, yellow laterally and ventrally; mesopleuron dark brown. Femora and tarsi dark brown. Forewing light brownish; colourless at base and at apex of cell r_1_; with brown submarginal band apically; radular areas along wing margin brown. Abdomen and terminalia almost black in male and light brown in female. Younger specimens with more extended light colour.

***Structure*.** Genal processes 0.7 times as long as coronal suture, blunt apically (Fig. 4D). Antenna 2.4 times as long as head width, relative length of flagellar segments from base to apex as 1.0: 1.1: 1.2: 1.3: 1.6: 1.5: 1.0: 0.4. Metatibia 0.6 times as long as head width. Forewing (Fig. 4H) 2.4–2.6 times as long as head width, 1.9–2.0 times as long as wide; broadly rounded apically. Terminalia as in Figs 6D, 7G, H, 8C. Male proctiger 0.4 times as long as head width, tubular in apical two-thirds, posterior lobe curved, pointing ventrad. Paramere longer than proctiger; in lateral view, lamellar; irregularly rounded apically; inner face with irregular, long setae, with a row of spaced marginal bristles along fore margin in apical half and group of thick bristles in apical quarter; with small, sclerotised hook apically. Distal segment of aedeagus shorter than proctiger; with moderately large, strongly curved apical dilatation. Female proctiger 1.2 times as long as head width, apex obliquely truncate, slightly upturned; circumanal ring 0.2 times as long as proctiger. Subgenital plate 0.7 times as long as proctiger, bearing dense bristles in apical half.

***Measurements*** (1 ♂, 1 ♀, in mm). Head width 0.82–0.86; antenna length 1.96–2.08; forewing length 1.98–2.20; male proctiger length 0.34; paramere length 0.36; length of distal segment of aedeagus 0.28; female proctiger length 1.00.

**Fifth instar immature** unknown.

#### Etymology.

Named after the Brazilian state of Roraima. A noun in apposition.

#### Host plant.

Unknown. Adults were collected on *Deguelia densiflora* (Fabaceae) which is a possible host.

#### Distribution.

Brazil (RR).

#### Affinities.

A member of the *Ciriacremum setosum* group, *C. roraima* sp. nov. shares the relatively short, apically blunt genal processes with *C. lanceolatum* sp. nov., from which it differs as indicated in the key. The antennae are shorter, the vein R_1_ of the forewing is longer, the paramere is lamellar rather than lanceolate and the apical dilatation of the aedeagus is smaller. *Ciriacremum roraima* sp. nov. differs from *C. setosum* in the shorter, apically blunt genal processes, the lamellar paramere (versus paramere with large anterior lobe) and the relatively longer female subgenital plate (about two-thirds as long as proctiger versus about half as long).

### ﻿ Ciriacremum

Taxon classificationAnimaliaHemipteraPsyllidae

sp. A

CE0273A5-9443-5044-85AB-FB0C10DF121E

Fig. 5A

#### Specimen figured on iNaturalist.

(https://www.inaturalist.org/observations/221943814) Brazil • 1 ♂; PA, Belém, Jurunas, Travessa Tupinambás; 1.4660°S, 48.4884°W; 10 Apr. 2024; photo taken by S. Dantas; no material.

#### Distribution.

Brazil (PA).

#### Affinities.

This species resembles *C. maederae* sp. nov. in its long, apically pointed or subacute genal processes and dark forewings, but differs by having a white forewing base. The species is new, but it is not formally named due to the absence of material (S. Dantas pers. comm.).

### ﻿ Ciriacremum

Taxon classificationAnimaliaHemipteraPsyllidae

sp. B

1807E7DA-045C-533E-A607-94A997CF6428

Fig. 5B, C

#### Material examined.

Brazil • 1 ♀; AM, 25 km NW Novo Airão; 30 Nov. 2000; U. Göllner leg.; at light; MNHU, dry mounted.

#### Distribution.

Brazil (AM).

#### Affinities.

This species resembles *C. pollicigerum* sp. nov. in its apically pointed or subacute genal processes and the transverse dark marginal band on the forewing, which is, however, indistinctly delimited (versus well defined in *C. pollicigerum*). The species is new, but it is not formally named due to the absence of male specimens.

##### ﻿Comments on other Neotropical *Ciriacremum* species

*Ciriacremum setosum* was originally described from a small series of males and females from Nicaragua (Crawford 1914). Later, Hodkinson and White (1981) also listed the species from Guyana based on a female housed in the NHMUK (https://data.nhm.ac.uk/dataset/56e711e6-c847-4f99-915a-6894bb5c5dea/resource/05ff2255-c38a-40c9-b657-4ccb55ab2feb/record/8593633). However, this is a misidentification and probably represents the same species as specimens from Venezuela housed in the MHNG (Aléné et al. 2007) and NHMUK (https://data.nhm.ac.uk/dataset/56e711e6-c847-4f99-915a-6894bb5c5dea/resource/05ff2255-c38a-40c9-b657-4ccb55ab2feb/record/8591790, https://data.nhm.ac.uk/dataset/56e711e6-c847-4f99-915a-6894bb5c5dea/resource/05ff2255-c38a-40c9-b657-4ccb55ab2feb/record/8592010). This latter species, representing a new taxon, differs from *C. setosum* in having a lamellar paramere (versus a broad paramere, bearing an anterior, narrowly rounded lobe), an apically obliquely truncate female proctiger (versus pointed), and a relatively long female subgenital plate. An unnamed species is also reported from Panama (Brown and Hodkinson 1988) and Colombia (Rendón-Mera et al. 2017), respectively. A fourth unnamed species is represented in the MNHU from Suriname (Brokopondo, Berg en Dal, 8 Oct. 1908, C. Heller leg.).

## ﻿Discussion and conclusions

With 36 valid species, *Ciriacremum* is species-rich in Africa (Hollis 1976; Aléné et al. 2007); this does not appear to be the case in the Neotropics, from where only five named species are currently known. The African species also appear to be more common and more widely distributed than the Neotropical species. Like the African species, the American species seem to develop on Fabaceae, although host information is available for only one species, *C. maederae* sp. nov.

The morphological similarity of the American species suggests that they constitute a monophyletic clade. There is no evidence that this is also true for the African species and, thus, the phylogenetic relationships of the two groups remain obscure. There is no cladistic analysis incorporating all ciriacremine genera. Using morphological characters, Hollis (1976) redefined *Ciriacremum* and *Kleiniella* Aulmann, and Brown and Hodkinson (1988) analysed the phylogenetic relationships of *Auchmerina* Enderlein, *Auchmeriniella* Brown & Hodkinson, *Euceropsylla* Boselli, *Heteropsylla* Crawford and *Manapa* Brown & Hodkinson. Percy et al. (2018) included in their molecular phylogenetic analyses *Auchmerina*, *Euceropsylla*, *Heteropsylla*, *Hollisiana* Burckhardt, Percy & Ouvrard, *Mitrapsylla* Crawford, and *Telmapsylla* Hodkinson. Based on similarities of details on the head and the male terminalia, we estimate that *Ciriacremum* and perhaps *Kleiniella* are closely related to *Mitrapsylla*.

Further fieldwork is necessary to find additional specimens of the currently unnamed species, to possibly find other undescribed species, and to evaluate the diversity of *Ciriacremum* in the New World. Additional material and analyses will help to elucidate the phylogeny which is a prerequisite for considerations on biogeography and host patterns.

## Supplementary Material

XML Treatment for Ciriacremum

XML Treatment for Ciriacremum lanceolatum

XML Treatment for Ciriacremum maederae

XML Treatment for Ciriacremum pollicigerum

XML Treatment for Ciriacremum roraima

XML Treatment for Ciriacremum

XML Treatment for Ciriacremum

## ﻿Additional information

### Use of AI

No use of AI was reported.

### Author ORCIDs

Daniel Burckhardt https://orcid.org/0000-0001-8368-5268

Dalva Luiz de Queiroz https://orcid.org/0000-0003-3556-9910

### Data availability

All of the data that support the findings of this study are available in the main text.
